# Household Factors Influencing Participation in Bird Feeding Activity: A National Scale Analysis

**DOI:** 10.1371/journal.pone.0039692

**Published:** 2012-06-28

**Authors:** Zoe G. Davies, Richard A. Fuller, Martin Dallimer, Alison Loram, Kevin J. Gaston

**Affiliations:** 1 Durrell Institute of Conservation and Ecology (DICE), University of Kent, Canterbury, Kent, United Kingdom; 2 School of Biological Sciences, The University of Queensland, St Lucia, Australia; 3 Division of Economics, Policy and Management Planning and Center for Macroecology, Evolution and Climate, University of Copenhagen, Copenhagen, Denmark; 4 Department of Animal and Plant Sciences, University of Sheffield, Sheffield, United Kingdom; 5 Environment and Sustainability Institute, University of Exeter, Penryn, Cornwall, United Kingdom; Institut Pluridisciplinaire Hubert Curien, France

## Abstract

Ameliorating pressures on the ecological condition of the wider landscape outside of protected areas is a key focus of conservation initiatives in the developed world. In highly urbanized nations, domestic gardens can play a significant role in maintaining biodiversity and facilitating human-wildlife interactions, which benefit personal and societal health and well-being. The extent to which sociodemographic and socioeconomic factors are associated with engagement in wildlife gardening activities remain largely unresolved. Using two household-level survey datasets gathered from across Britain, we determine whether and how the socioeconomic background of a household influences participation in food provision for wild birds, the most popular and widespread form of human-wildlife interaction. A majority of households feed birds (64% across rural and urban areas in England, and 53% within five British study cities). House type, household size and the age of the head of the household were all important predictors of bird feeding, whereas gross annual household income, the occupation of the head of the household, and whether the house is owned or rented were not. In both surveys, the prevalence of bird feeding rose as house type became more detached and as the age of the head of the household increased. A clear, consistent pattern between households of varying size was less evident. When regularity of food provision was examined in the study cities, just 29% of households provided food at least once a week. The proportion of households regularly feeding birds was positively related to the age of the head of the household, but declined with gross annual income. As concerns grow about the lack of engagement between people and the natural environment, such findings are important if conservation organizations are successfully to promote public participation in wildlife gardening specifically and environmentally beneficial behaviour in society more generally.

## Introduction

The prospects for maintaining large terrestrial land parcels for conservation that are relatively undisturbed by human activities have already been lost for much of the world [1]–[2]. Additional conservation measures are therefore being applied in the wider landscape, outside of protected areas, in order to preserve species. Such initiatives often have many added benefits including supporting ecosystem function [3], augmenting ecosystem service provision [4]–[5], and enhancing human health and well-being [6]–[7]. As a greater proportion of the world’s human population comes to live in cities [8], the advantages of extending management to enhance biodiversity within urban and residential areas are increasingly being recognized, not least given that this is where the majority of the human population will experience interactions with wildlife in such highly urbanized societies [9]–[10]. Indeed, evidence of the benefits to the human population of experiencing and interacting with wildlife and the natural world is accruing rapidly (e.g., [11]–[13]). The personal and societal gains are diverse, but include added health benefits when exercise is carried out in natural environments [14]–[15], improvements in self-reported general health [16]–[18], enhanced longevity [19], stress-relief [20], reduced mental fatigue [21], increased degree of social interaction [22] and lower crime rates [23].

A variety of strategies have been suggested to ameliorate pressures on the ecological condition of residential environments. These include creating green networks and corridors [24]–[25], developing urban forests [26]–[27], improving the management of public parks (e.g., [28]–[29]), and encouraging householders to participate in ‘wildlife gardening’ activities (e.g., [30]). Wildlife gardening can be broadly defined as any action conducted in a domestic garden intended to increase its suitability for species, including the provision of a diversity of resources (e.g., food, breeding and overwintering sites) [31]. One of the attractions of such an approach has been the potential for mass participation by individual households; gardens are intensively managed habitats, in which private landowners may invest substantial amounts of both time and money. Indeed, the UK garden retail market is currently worth £4.6 billion [32] and, in 2005, a national time use survey revealed that 13% of adults engage in gardening, spending on average 17 minutes per day doing so [33].

Although gardens are managed by individual households, their importance for biodiversity conservation and ecosystem service provision through mass participation is recognized not only by the research community (e.g., [34]–[36]), but also by local (e.g., [37]–[39]) and national (e.g., [40]–[42]) authorities. An understanding of how participation in wildlife gardening activities may vary with the socioeconomic characteristics of individual households is important if conservation organizations are to promote further public engagement in wildlife gardening activities, and to develop strategies to increase awareness of environmentally beneficial behaviour in society more widely [43]–[44].

In both the UK and US, the most popular wildlife gardening activity is feeding wild birds [42]–[43]. Although a number of studies have explored both the positive and negative effects of food provision on bird populations and communities (e.g., [34], [47]–[53]), the socioeconomic factors underpinning such human-wildlife interactions within domestic gardens have seldom been investigated explicitly (but see [54] for a single region study, and [50] for an analysis resolved only to the neighbourhood level).

In this paper, we develop *a priori* hypotheses regarding the relationships between the sociodemographic and socioeconomic status of individual households, whether they engaged in bird feeding activities and how regularly food was provided (Table 1). Based on previous research in related areas, we focus on six fundamental household characteristics, for which data are straightforward to obtain (thus allowing conservation groups wishing to launch initiatives to increase public uptake of wildlife gardening to build on the outcomes of this study), and determine whether they can be used to predict involvement in bird food provision. Although our hypotheses are informed by the existing primary literature, the majority of these studies examine correlative associations between socioeconomic status and measures of biodiversity, rather than on household participation in activities that could support biodiversity.

**Table 1:** A summary of the predicted relationship between each household characteristic and the prevalence of food provision for wild birds, based on the findings of previous studies investigating various human-wildlife interactions.

Household characteristic	Prediction	Literature supporting the choice of household characteristic and/or prediction
*Household Status*	Feeding more likely in owned, rather than rented, households	Luck et al. (2009)
*House Type*	Feeding more likely in increasinglydetached house types.	Gaston et al. (2007); Loram et al. (2007)
*Age of Householder*	Feeding more likely where the head of thehousehold is older	Lepczyk et al. (2004); Booth et al. (2009); Natural England (2010)
*Household Size*	Feeding is influenced by the number ofpeople in the household.	Lepczyk et al. (2004)
*Gross Annual Household Income*	Feeding more likely in households withhigher annual income	Hope et al. (2003); Kinzig et al. (2005); Luck et al. (2009); Strohbach et al. (2009)
*Occupation/Employment Status of Householder*	Feeding more likely in occupations ofpeople in higher socio-economic groups	Fuller et al. (2008); European Commission (2010)

1. *Household Status*. At a neighbourhood-level, home ownership in Australia was positively correlated with abundance of nectar-rich plants and native trees, and negatively associated with impervious surface cover [55]. The authors suggested that home owners are likely to have a greater attachment to their land and property, and are therefore more prone to investing in garden maintenance that could be beneficial for wildlife. We thus hypothesize that home owners will be more likely to undertake bird feeding activity.

2. *House Type*. Garden area has been found to be positively correlated with participation in bird feeding activity [46]. Given that housing type (in Britain, whether a house is detached, semi-detached, terraced or is a flat) is a reliable surrogate measure of garden area [56], we predict that the bird food provision will be greater as houses become progressively more detached.

3. *Age of Householder.* There is concern among policy-makers [57] about the decline in human-wildlife interactions, especially amongst children and young adults [58]–[59]. Older members of the public are more likely to engage in activities related to the natural environment in general [60]. In a single region study, older householders in Michigan were more engaged in bird feeding [54], a trend that, although not formally tested, has also been reported for the US as a whole. We therefore anticipate that bird feeding will be positively related to the age of the householder.

4. *Household Size*. In their study region, Lepczyk et al. [54] failed to find an association between the number of people in a household and participation in wildlife gardening activities. However, this does not preclude the possibility household size may influence the provision of food for birds at a national-scale, if only on the grounds that larger households may be more likely to contain one or more individuals interested in undertaking such activities.

5. *Gross Annual Household Income*. Household income was positively associated with measures of vegetation cover in Australia [55], while family income explained spatial variation in plant diversity across different neighbourhoods in Phoenix, US [61]–[62]. Similarly, in Germany, bird species richness was greater in neighbourhoods where the average income of residents was high [63]. In the UK, the proportion of households providing food for birds was negatively related to an index of socioeconomic deprivation [50]. As such, we hypothesize that the prevalence of bird feeding activity will increase with gross annual household income, not least because the cost of purchasing both bird food and feeding equipment may discourage lower income groups from participating in the activity.

6. *Occupation/Employment Status of Householder*. Across Europe, the proportion of people who reported making personal efforts to protect biodiversity varied according to occupation/employment status [64]. We thus predict that the occupation of the head of the household will influence whether or not food is provided for birds.

In addition to testing these hypotheses, we also examine how the level of participation in bird feeding varies for each household characteristic identified as being an important predictor of engagement. This is the first time that such trends at an individual household-level have been formally assessed at a nationwide scale.

## Methods

**Figure 1:**
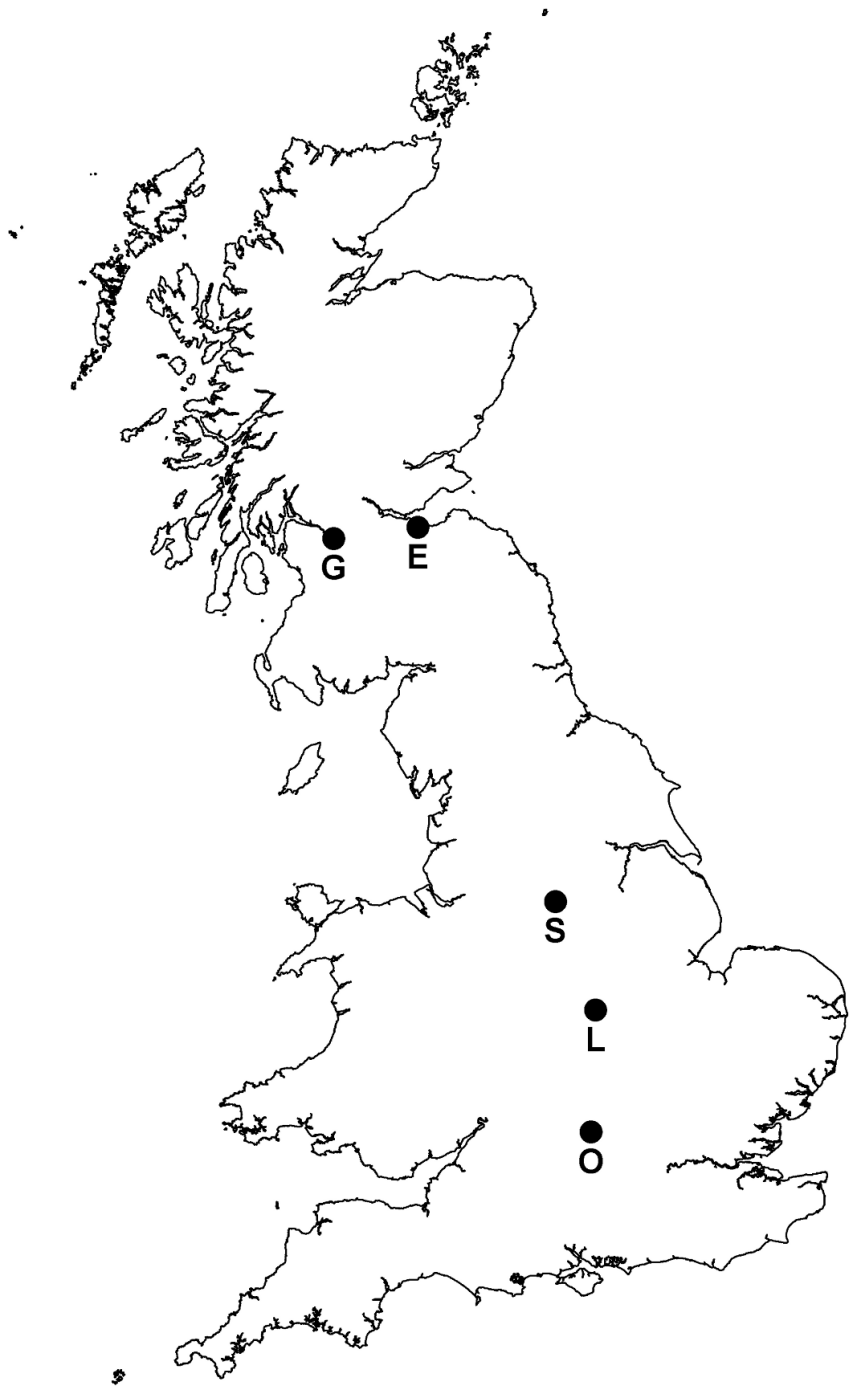
The location of the five British cities (E, Edinburgh; G, Glasgow; L, Leicester; O, Oxford; S, Sheffield) sampled during the CityForm survey, the data from which were subsequently used to investigate whether and how the socioeconomic and sociodemographic background of a household influences participation in wild bird feeding activity.

We carried out this study using two household surveys. The first comprises data collected on participation in bird feeding activities gathered in England, covering both rural and urban areas across the country, and the second examines food provision for birds within five major British cities (Figure 1). Using these complementary datasets allows us to contrast households situated within urban areas specifically and the general population as a whole.

### Survey of English Housing

The Survey of English Housing (SEH) is an annual interview-based survey completed by approximately 20,000 households (Table 2), conducted for the UK government’s Department for Communities and Local Government by the National Centre for Social Research. All data were gathered in accordance with UK government confidentiality and data protection regulations and were fully anonymised prior to use in this study [65]. The main purpose of the survey is to gather reliable information on the main features of each household and the attitude of the respondent in relation to their personal housing circumstances. In its entirety, the survey consists of approximately 800 questions, comprising a core of factual questions that remain largely unchanged from year to year (e.g., regarding factors such as whether the respondent owns or rents the property, living costs and housing history), in addition to a set of questions on opinions and intentions that are revised annually (see http://www.esds.ac.uk/for details). The surveyed households are chosen at random from within stratified groupings based on Government Office Regions and socioeconomic status.

**Table 2:** Response rates for the Survey of English Housing (SEH) and CityForm questionnaire.

Survey name	Area covered	No. of households approachedto participate in the survey	No. of completed questionnaires	Response rate (%)
SEH	England	29,786	19,913	67
CityForm	Edinburgh	2593	1074	41
CityForm	Glasgow	2533	741	29
CityForm	Leicester	2072	633	31
CityForm	Oxford	2274	792	35
CityForm	Sheffield	2421	1141	47

A full breakdown of CityForm questionnaire response rates for the three study areas in each city can be found in Gaston et al. (2007).

The 2001/02 survey [66] included a small set of questions investigating the degree to which households participate in wildlife gardening activities. It represents the most recent national-level survey data pertaining to wildlife gardening, thereby allowing an in depth investigation of the socioeconomic characteristics of households that undertake bird feeding activities (as opposed to previous neighbourhood-level analyses [50]). Respondents were asked whether the household provided food for birds and/or had a bird feeder or table. Elsewhere in the questionnaire, respondents were asked to indicate whether they had access to a private, shared or communal garden, patio or yard, or none of these. Over two-thirds of surveyed households completed the questionnaire (Table 2).

### CityForm Questionnaire

The CityForm questionnaire survey was conducted in 2005, as part of a large consortium research project investigating social, economic and environmental urban sustainability (see [46], [67] for full details of the survey methodology, and Table 2 for response rates). Data were collected from five cities across Britain: Edinburgh, Glasgow, Leicester, Oxford and Sheffield (Figure 1). Within each city, addresses were selected from three different study sites representing a city centre location, an outer suburban site and a site situated in between the two. Sites were selected to represent the range of urban form within each city; formal comparisons between cities are therefore not appropriate [67] and all data from the CityForm survey are analyzed together. The questionnaire contained 50 questions relating to the aims of the wider consortium project, and thus the four questions on wildlife gardening used in this study formed only a small part of the survey. This structure minimized the potential biases associated with the level of interest that questionnaire recipients had in wildlife and/or gardening. All data for the CityForm survey were gathered and stored anonymously. Appropriate institutional ethics procedures were followed.

As with the SEH, respondents were asked to indicate whether they had access to a private garden, shared/communal garden, patio or yard, roof terrace/large balcony or none of these. Respondents were then asked to indicate how regularly food was provided for birds by household members, choosing one option from the following categories: daily, weekly, monthly, less than monthly, or never.

### Data Extraction and Standardisation

We extracted data from both surveys relevant to the six hypotheses: whether the household was owned or rented (*Household Status*), the type of house (*House Type*), the age of the head of the household (*Age of Householder*), the number of people resident at the property (*Household Size*), gross annual income for the household (*Gross Annual Household Income*) and the nature of employment of the head of the household (*Occupation of Householder* for the SEH, and *Employment Status of Householder* for the CityForm questionnaire). The information was then re-coded into categorical responses which were comparable between the SEH and CityForm questionnaire (Table S1, S2).

**Table 3:** The probability (*k*) of each household characteristic being an important predictor (i.e., better than random; highlighted in bold) of the level of participation in bird food provision, for three datasets collected as part of the Survey for English Housing (SEH) and CityForm questionnaire.

Household characteristic	SEH (feed birds:yes or no)	CityForm (feed birds:yes or no)	CityForm (feed birds:regularly or irregularly)
*Household Status*	0.337	0.288	0.462
*House Type*	**1.000**	**1.000**	0.498
*Age of Householder*	**1.000**	**1.000**	**1.000**
*Household Size*	**0.923**	**0.999**	0.281
*Gross Annual Household Income*	0.280	0.493	**0.854**
*Occupation/Employment Status of Householder*	0.352	0.568	0.754
Random Explanatory Variable (95% CI)	0.269–0.810	0.270–0.710	0.270–0.804

### Statistical Analyses

Prior to conducting the statistical analyses, we removed households reporting no access to an outside space from both the SEH and CityForm datasets, as these households would not be able to participate in bird feeding activity regardless of their sociodemographic or socioeconomic background. The data obtained in the CityForm survey regarding whether households provided bird food were consolidated into binomial responses (yes or no) to allow for direct comparison with the SEH findings. In addition, we constructed a third dataset by grouping the CityForm data pertaining to how regularly food was provided for birds into two response categories: regularly (households feeding birds daily or weekly and thus providing significant levels of food) and irregularly (households providing bird food on a monthly or less than monthly basis). This allowed us to adopt the same statistical approach to investigate the relationship between household characteristics and regularity of food provision. All statistical analyses were carried out using R (release version 2.10.1 [68]).

For each of the three datasets, multiple colinearity between the household characteristics was investigated and found to be within accepted norms [69]. Correlation matrices showed that the relationships between the household characteristics left much of the variation in the data unexplained (with a maximum r_s_ recorded of 0.53), and Variance Inflation Factors (VIFs) among all six variables were not sufficient to preclude multivariate analysis.

To determine which of the six household characteristics were important predictors (i.e., better than random) of the level of participation in bird feeding activity within each dataset, we used the Information Theoretic approach [70]. All possible subsets of the household characteristics were modelled using logistic regression, with the binomial response to bird feeding as the dependent variable (yes or no for SEH and CityForm, and regularly or irregularly for CityForm). For each individual model within the complete set (which consisted of 64 models in total), we calculated the Akaike’s Information Criterion (AIC) and the Akaike weight (*w_i_*). The best fitting model was defined as that with the lowest AIC. The probability of each household characteristic appearing in the best fitting model (*k*) could then be estimated. However, as poor predictor variables do not necessarily have selection probabilities close to zero, a single randomly generated explanatory variable, unrelated to the response variable, was added to the existing dataset [71]. Five hundred model sets were subsequently generated and *k* was estimated for the random explanatory variable. Household characteristics that were important predictors of participation in bird feeding activity had a value for *k* which fell outside the 95% confidence intervals for the random explanatory variable.

**Table 4:** Analysis of deviance models (GLM with binomial errors and logit link function) used to detect differences between the proportions of households providing food for birds across household characteristic categories, within the Survey for English Housing (SEH) and CityForm questionnaire.

Dataset	Household Characteristic	n	κ^2^	d.f.	P
SEH (feed birds: yes or no)	*House Type*	17965	1109.7	0,5	<0.001
	*Age of Householder*	18042	1039.8	0,6	<0.001
	*Household Size*	18042	223.5	0,6	<0.001
CityForm (feed birds: yes or no)	*House Type*	3781	493.6	0,5	<0.001
	*Age of Householder*	3784	234.4	0,6	<0.001
	*Household Size*	3705	104.7	0,6	<0.001
CityForm (feed birds: regularly or irregularly)	*Age of Householder*	2014	116.6	0,6	<0.001
	*Gross Annual Household Income*	1527	35.6	0,6	<0.001

Mixed models were also developed that included UK government region and city as a random factor for the SEH and CityForm datasets respectively. For the CityForm analyses, including city led to an increase in AICc compared to the fixed effect only models. For the SEH models, including region as a random factor led to the mixed models being more parsimonious (lower AICc). However, there was no change in the form of the relationships between the explanatory and response variables and only minimal alterations in the parameter estimates. In order to retain a consistent analytical approach across the three datasets, we therefore present results from the fixed effect only models.

**Figure 2:**
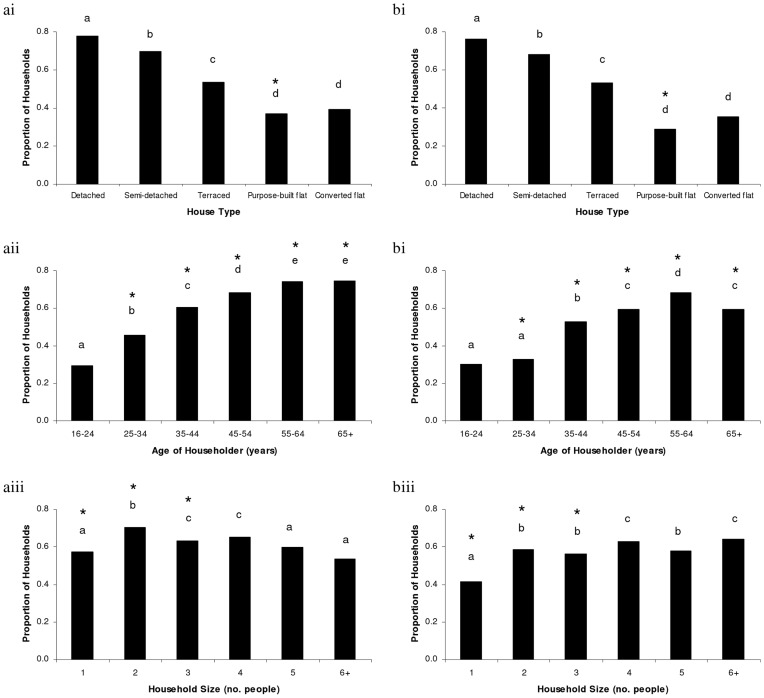
The proportion of households recorded within the a) Survey of English Housing and b) CityForm questionnaire that participated in food provision for wild birds in the outside space associated with their dwelling, for the three household characteristics that were found to be significant predictors of bird feeding activity: (i) *House Type*, (ii) *Age of Householder*, and; (iii) *Household Size*. Letters denote, within each household characteristic dataset, which categories have significantly different proportions of households feeding birds. Stars indicate a significant difference in the proportion of households providing food for birds between the SEH (wider population) and CityForm (urban only) surveys for comparable categories.

For every household characteristic identified as an important predictor in each dataset, differences in the proportion of households engaged in bird feeding activity were investigated between categories using a generalized linear model (GLM) with binomial errors and logit link function. The resulting GLM was an analysis of deviance, analogous to an ANOVA, and post-hoc contrasts [72] were used to determine which categories significantly differed. Finally, we explored differences in the proportion of households providing food for birds for each household characteristic category, but between the SEH and CityForm questionnaire datasets, using a 2-sample test for equality of proportions with continuity correction [72].

**Figure 3:**
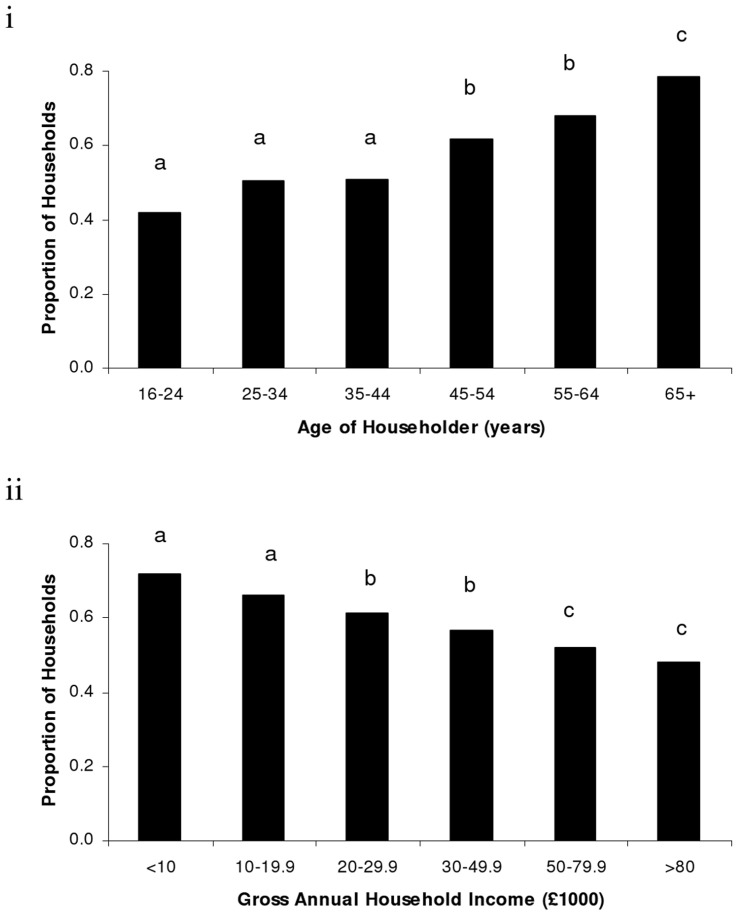
The proportion of households recorded within the CityForm questionnaire that regularly (i.e., at least once a week) provided food for birds in the outside space associated with their dwelling, for the two household characteristics that were found to be significant predictors of participation in regular bird feeding activity: (i) *Age of Householder*, and; (ii) *Gross Annual Household Income*. Letters denote, within each household characteristic dataset, which categories have significantly different proportions of households feeding birds.

## Results

### Household Access to a Garden

Of the 19913 households surveyed in the 2001/02 SEH, 91% (n = 18042) reported access to a shared or communal garden, yard or patio area. This result was comparable to that recorded by the CityForm questionnaire, which found that 87% (n = 3819) of the 4381 households that responded to the survey had a garden, yard or patio associated with the dwelling.

### Household Participation in Bird Feeding Activity

In the SEH, over 64% (n = 11620) of households provided food for birds and/or had a bird feeder or table in their garden area. In contrast, only 53% (n = 2027) of households that completed the CityForm questionnaire were participating in bird feeding activity in their outside space. For both the SEH and CityForm datasets, the same three household characteristics were better than random predictors of participation in bird feeding activity: *House Type*, *Age of Householder* and *Household Size* (Table 3).

We subsequently examined the differences between categories within each of these household characteristics (Table 4). The trends for *House Type* were the same in both surveys, with significantly higher proportions of households feeding birds when living in progressively more detached properties (Figure 2). In the SEH, the proportion of households providing food for birds increased significantly with each category for *Age of Householder*, until 55 years and over. The results for the CityForm questionnaire were broadly similar, with a lower prevalence of bird feeding activity occurring in the two youngest age categories; the proportion of households feeding birds then increased significantly with successive age categories, before tailing off with the 65+ age bracket. The patterns for *Household Size* were significant but less systematic, both within and between the two survey datasets. The only trend common to both the SEH and CityForm surveys was that households consisting of just a single individual were less likely to be engaged in bird feeding activity, relative to larger households.

### The Regularity of Food Provision by Households

When we investigated the regularity of food provision for birds using the CityForm dataset, only 64% (n = 1291) of the 2027 households feeding birds were found to be doing so on a regular basis (i.e., at least once a week), which equates to 29% of all respondent households with access to an outside space. *Age of Householder* and *Gross Annual Household Income* were the only two predictors of how regularly households provided bird food that were better than random (Table 3).

Significant differences between categories for both of these household characteristics were apparent (Table 4). For *Age of Householder*, the smallest proportions of households feeding birds on a regular basis were in the less than 44 year old age categories. The proportions in the 45 to 64 year old age groups were higher, but the greatest proportion of households providing food for birds regularly was in the 65+ age bracket. The regularity of bird feeding decreased with increasing *Gross Annual Household Income*. Households with an income of less than £20,000 were more likely to be providing food for birds at least once a week, than those households with an income of between £20,000 and £49,999. The lowest proportions of households feeding at regular intervals all had a gross annual income in excess of £50,000 (Figure 3).

## Discussion

Across England, 64% of households with access to a domestic garden provided food for birds. Although the proportion of households engaging in food provision within their garden was smaller in the urban survey, approximately half of the households (53%) in five British cities were still participating in the activity. These estimates are likely to be robust as the households taking part in the surveys were selected from the general population and were not necessarily bird enthusiasts (in contrast to most garden bird monitoring schemes that collect data on food provision, such as the British Trust for Ornithology’s Garden BirdWatch [73]). Indeed, the questions pertaining to gardens and bird feeding comprised only a small fraction of the entire survey, for both the SEH and CityForm questionnaires, ensuring that the probability of a household responding was independent of the head of the household’s level of interest in gardening and/or biodiversity.

Of the six sociodemographic and socioeconomic household characteristics identified as potential factors that may influence the likelihood of participation in wildlife gardening activity, we found that *House Type*, *Age of Householder* and *Household Size* were important predictors of engagement in food provision, both across England and within the study cities. The patterns of household participation for each of these characteristics were broadly consistent between the SEH and the CityForm questionnaire datasets.

In both the surveys, the proportion of households feeding birds increased as households became progressively more detached and as the age of the head of the household increased. In agreement with our findings, a study investigating landowner activities along a rural-urban gradient in southeast Michigan [54] established that older people were more likely to provide food for birds. Similarly, the prevalence of bird feeding in households was not related to the occupation of the head of the household. Yet, in contrast to our results, Lepczyk et al. [54] found that there was no significant difference in the number of people living at a dwelling and whether householders did or did not put food out for wild birds.

Using the CityForm dataset, we found that 64% of the households feeding birds in their urban garden areas did so on a frequent basis (i.e., at least once a week), which equates to 29% of all households with access to an outside space. Cowie and Hinsley [74] assessed patterns in bird feeding in suburban gardens in Cardiff and found that 79% of households provided food for birds during the winter, but just 56% did so regularly (in this instance on a daily basis). Likewise, during the summer months, 52% of households provided food for birds, yet 19% of this activity was only occasional. Bird feeding, though prevalent, is therefore an infrequent activity.

In the UK and other countries, private landowners are frequently encouraged to provide food for birds, in order to enhance the survival of avian populations and augment the ecosystem services they provide (e.g., [36], [75]–[79]). Here we find that 64% of households in England with access to a domestic garden feed wild birds. In the US, 23% of citizens engage in bird feeding at home [45], and between 25 and 57% of households in Australia put out food for avian visitors [51]. Over $3.4 billion is spent on bird food annually in the US alone [45], and the global market for bird seed is growing at a rate of 4% per year [80].

Nonetheless, the ecological impacts of this particular wildlife gardening activity are highly controversial and are likely to vary between countries (see [51] for a review). Research suggests that domestic gardens can play an important role in supporting avian populations by increasing the availability of food resources (e.g., [34], [49]–[50], [81]–[83]), and feeding experiments have documented significant positive effects on the abundance, condition and productivity of specific species at various scales (e.g., [47]–[48], [84]–[88]). However, opponents to food provision stress that there are many potentially detrimental effects that are yet to be fully investigated. These could include a reliance on an unpredictable resource, a reduction in diet quality, loss of natural foraging behaviours [87], [89], the spread of disease [52], [90], loss of reproductive output [53], increased predation risk at feeders as a result of higher predator density [51], and an increase in the number and abundance of exotic species [34], [83]. Further research is therefore required to understand better how the spatial distribution, temporal frequency and quality of food provision for wild birds in domestic gardens may influence the conservation value of the activity. This is particularly important given that year-round bird feeding is advocated by UK NGOs [91].

Here, we draw attention to socioeconomic characteristics that underlie one set of interactions between human society and biodiversity. Research in this area, although infrequent in the literature, is of particular relevance to statutory agencies and non-governmental organizations that are currently involved in endorsing biodiversity conservation actions to private landowners. For instance, the UK government’s Department for Environment, Food and Rural Affairs (Defra) has used ‘the proportion of households undertaking wildlife gardening’ as one of their urban biodiversity targets for England [40]–[42]. The clear trends evident in this study could be used to inform strategies aimed at raising awareness in the general public of the benefits of interacting with nature within domestic gardens, by identifying key social groups to be targeted. For example, city councils and local authorities could use planning regulations and tax incentives directed towards particular income groups or housing types to increase participation in wildlife-friendly gardening activities [36]. Alternatively, as bird feeding cannot be easily directed by government, a community-led approach could be taken by NGOs to encourage a greater participation in food provision, under existing initiatives such as the RSPB’s “Homes for Wildlife” scheme in the UK or the USA National Wildlife Federation Backyard Habitat Certification Scheme, which are increasingly targeted at particular sectors of society. In following these strategies, we further propose two approaches that could be adopted: (i) to focus on sociodemographic and socioeconomic groups where low proportions of households are undertaking such activities and, therefore, where the most impact could be made, or; (ii) to target sociodemographic and socioeconomic groups where high proportions of households participate in activities and where additional involvement may more readily be accomplished.

As the general public get progressively more disinterested in nature [9], [58]–[59], finding creative and pertinent mechanisms through which to promote the integration of conservation action into everyday life is vital in order to both support biodiversity, and enhance human health and well-being [43]–[44], [57]. An appreciation of the sociodemographic and socioeconomic background of the households to be targeted within a campaign, as acquired by this study, will allow conservation groups to tailor their advice accordingly and communicate more effectively with their intended audience.

## Supporting Information

Table S1 Survey of English Housing questions pertaining to the six sociodemographic household characteristics that were identified as those that may influence the likelihood of households providing supplementary food for birds. Superscript numbers indicate how data was re-categorized where applicable.
(DOCX)

Table S2 CityForm survey questions pertaining to the six sociodemographic household characteristics that were identified as those that may influence the likelihood of households providing supplementary food for birds. Superscript numbers indicate how data was re-categorized where applicable.
(DOCX)
