# Supplementary material for: Household Factors Influencing Participation in Bird Feeding Activity: A National Scale Analysis
Source: PLoS One. 2012 Jun 28;7(6):e39692. doi: 10.1371/journal.pone.0039692 (PMC3386264; doi:10.1371/journal.pone.0039692)
Supplement: Table S1 — Survey of English Housing questions pertaining to the six sociodemographic household characteristics that were identified as those that may influence the likelihood of households providing supplementary food for birds. Superscript numbers indicate how data was re-categorized where applicable. (DOCX) [file pone.0039692.s001.docx]

**Table S1:** Survey of English Housing questions pertaining to the six sociodemographic household characteristics that were identified as those that may influence the likelihood of households providing supplementary food for birds. Superscript numbers indicate how data was re-categorized where applicable.

| **Household characteristic** | **Relevant survey question** | **Possible responses (*type of data*)** | **Categorical responses for analysis (*exclusions*)** |
| --- | --- | --- | --- |
| *Household Status* | In which of these ways do you occupy the accommodation? | ^1^Own outright, ^1^buying it with the help of a mortgage or loan, ^2^rent it, ^2^live here rent free (excluding squatting), squatting (*categorical*) | ^1^Own, ^2^rent (*squatting due to low sample size and no comparable category in the CityForm questionnaire*) |
| *House Type* | Is the accommodation a …? | ^1^Detached house/bungalow, ^2^semi-detached house/bungalow, ^3^terraced or end of terrace house/bungalow, ^4^purpose built flat/maisonette, ^5^converted flat/maisonette, house boat, caravan (*categorical*) | ^1^Detached, ^2^semi-detached, ^3^terraced or end of terrace, ^4^purpose built flat/maisonette, ^5^converted flat/maisonette (*house boat and caravan due to low sample size and no comparable category in the CityForm questionnaire*) |
| *Age of Householder* | What was your age last birthday? | Any (*continuous*) | 16-24, 25-34, 35-44, 45-54, 55-64, 65 or over |
| *Household Size* | How many people live in your household? | Any (*continuous*) | 1, 2, 3, 4, 5, 6 or over |
| *Gross Annual Household Income* | What is the total income of the whole household before deductions for income tax, National Insurance etc.? | Any (*continuous*) | Under £10000, £10000-£19999, £20000-£29999, £30000-£49999, £50000-£79999, £80000 or more |
| *Occupation of Householder* | What was your last or current job? (supplementary information was provided in order to correctly assign householders to the appropriate category) | Higher managerial, higher professional, lower professional/higher technical, lower managerial, higher supervisory, intermediate, small employers, own accounts workers, lower supervisory, lower technical, semi-routine, routine (*categorical*) | Higher managerial, higher professional, lower professional/higher technical, lower managerial, higher supervisory, intermediate, small employers, own accounts workers, lower supervisory, lower technical, semi-routine, routine |
