# Supplementary material for: Household Factors Influencing Participation in Bird Feeding Activity: A National Scale Analysis
Source: PLoS One. 2012 Jun 28;7(6):e39692. doi: 10.1371/journal.pone.0039692 (PMC3386264; doi:10.1371/journal.pone.0039692)
Supplement: Table S2 — CityForm survey questions pertaining to the six sociodemographic household characteristics that were identified as those that may influence the likelihood of households providing supplementary food for birds. Superscript numbers indicate how data was re-categorized where applicable. (DOCX) [file pone.0039692.s002.docx]

**Table S2:** CityForm survey questions pertaining to the six sociodemographic household characteristics that were identified as those that may influence the likelihood of households providing supplementary food for birds. Superscript numbers indicate how data was re-categorized where applicable.

| **Household characteristic** | **Relevant survey question** | **Possible responses (*type of data*)** | **Categorical responses for analysis (*exclusions*)** |
| --- | --- | --- | --- |
| *Household Status* | Do you or other household member own your home? | ^1^Own outright/own with a mortgage or loan, pay part mortgage part rent, ^2^rent from public sector, ^2^rent from private sector (*categorical*) | ^1^Own, ^2^rent (*pay part mortgage part rent due to low sample size and no comparable category in the SEH*) |
| *House Type* | What type of accommodation do you live in? | ^1^Detached house/bungalow, ^2^semi-detached house/bungalow, ^3^terraced or end of terrace house/bungalow, ^4^purpose built flat/maisonette, ^5^converted flat/maisonette, dwelling above a shop/office (*categorical*) | ^1^Detached, ^2^semi-detached, ^3^terraced or end of terrace, ^4^purpose built flat/maisonette, ^5^converted flat/maisonette (*dwelling above a shop/office due to low sample size and no comparable category in the SEH*) |
| *Age of Householder* | To which of these age groups does the head of the household belong? | 16-24, 25-34, 35-44, 45-54, 55-64, 65 or over (*categorical*) | 16-24, 25-34, 35-44, 45-54, 55-64, 65 or over |
| *Household Size* | How many people are in your household? | Any (*continuous*) | 1, 2, 3, 4, 5, 6 or over |
| *Gross Annual Household Income* | What is your gross annual household income? | Under £10000, £10000-£19999, £20000-£29999, £30000-£49999, £50000-£79999, £80000 or more (*categorical*) | Under £10000, £10000-£19999, £20000-£29999, £30000-£49999, £50000-£79999, £80000 or more |
| *Employment Status of Householder* | Which of the following best describes the economic status of the householder? | Employed full-time (more than 30 hours a week), employed part-time (less than 30 hours a week), self-employed/freelance, unemployed/seeking work, retired, at home/family carer, full time student at college/university, long-term sick/disabled (*categorical*) | Employed full-time, employed part-time, self-employed/freelance, unemployed/seeking work, retired, at home/family carer, full time student at college/university, long-term sick/disabled |
